# Pylephlebitis and septic thrombosis of the inferior mesenteric vein secondary to diverticulitis

**DOI:** 10.1590/0100-3984.2017.0046

**Published:** 2018

**Authors:** Rodolfo Mendes Queiroz, Fernando Dias Couto Sampaio, Pedro Eduardo Marques, Marcus Antônio Ferez, Eduardo Miguel Febronio

**Affiliations:** 1 Documenta - Hospital São Francisco, Ribeirão Preto, SP, Brazil.; 2 Hospital São Francisco - Centro de Terapia Intensiva, Ribeirão Preto, SP, Brazil.

*Dear Editor,*

A 74-year-old diabetic male patient presented with a 15-day history of abdominal pains and episodes of fever. Laboratory findings included leukocytosis and discretely increased C-reactive protein, as well as aspartate aminotransferase (AST) and alanine aminotransferase (ALT) at the upper limits of normality. Non-contrast-enhanced computed tomography (CT) of the abdomen showed signs suggestive of an inflammatory process with sigmoid diverticulosis (Figure 1A). The patient presented clinical worsening, was unresponsive to antibiotic therapy, and evolved to jaundice in one day. Additional laboratory tests showed increases in leukocytosis, C-reactive protein, AST, ALT, and total bilirubin. A blood culture showed growth of *Citrobacter* spp., *Streptococcus* spp., and *Klebsiella* spp. A contrast-enhanced CT scan of the abdomen demonstrated thrombi and gas in the portal venous system (Figure 1B), spleno-mesenteric junction (Figure 1C) and inferior mesenteric vein (Figure 1D). Subtotal colectomy was performed, and the pathology study confirmed an acute inflammatory process with sigmoid diverticulosis and an incidental finding of a small cecal adenocarcinoma. The antibiotics were changed, and parenteral anticoagulation was started. The patient evolved to clinical improvement, being discharged after one month.

**Figure 1:**
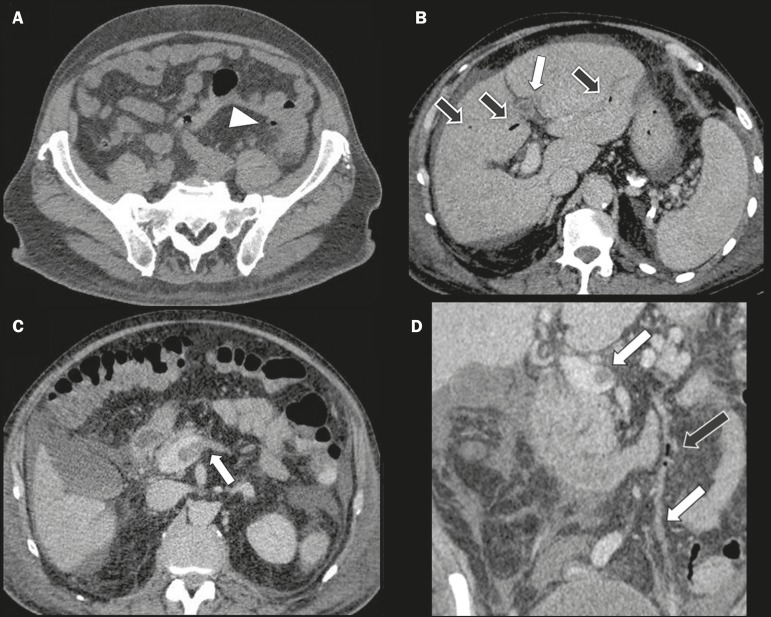
A: Non-contrast-enhanced abdominal CT of the patient at admission, showing increased density of mesenteric fat around the sigmoid colon (arrowhead), where some colonic diverticula were also present, suggesting an inflammatory process. B,C,D: Intravenous contrast-enhanced CT, acquired in the portal phase, after clinical worsening of the patient, showing thrombi (white arrows) in the left branch of the portal vein (B), spleno-mesenteric junction (C,D) and inferior mesenteric vein (D), together with gaseous foci (black arrows) in the intrahepatic portal venous system (B) and inferior mesenteric vein (D), as well as ascites.

Pylephlebitis, which is characterized by septic thrombosis of the portal vein or its branches^(1-3)^, has an annual incidence of 0.37-2.7 cases per 100,000 inhabitants per year^(4,5)^. It occurs in 0.16% of patients with intra-abdominal infections^(4)^. It typically affects individuals between 40 and 65 years of age, and 60-70% of the affected individuals are male^(4-6)^. The main causes include diverticulitis (in 19-30% of cases), pancreatitis (in 5-31%), appendicitis (in 2-19%), infections of the biliary tract (in 3-14%), and inflammatory bowel disease (in 2-6%)^(4-7)^, as well as umbilical catheterization and omphalitis in neonates^(3)^. Risk factors for pylephlebitis include the following^(4-6)^: a history of surgery, seen in 29-37% of patients; smoking, seen in 29%; malignancies, seen in 6-17%; immunosuppression, seen in 14%; blood dyscrasias; alcoholism; and steroid use.

The clinical presentation of pylephlebitis is nonspecific, common symptoms being fever, abdominal pain, nausea, diarrhea, and anorexia; however, a presentation of jaundice accompanied by fever and abdominal pain should raise the suspicion of the disease^(4-6)^. Laboratory findings include leukocytosis (in 80% of cases), positive culture in blood or tissues (in 44-88%), elevated liver enzymes (in 40-69%), and total bilirubin (in 55%). From cultures, a single microorganism is isolated in 47% of cases and multiple microorganisms are isolated in 44%, the most common being anaerobic, gram-negative bacteria. The pathogens typically identified include *Escherichia coli*, *Streptococcus* spp., *Bacteroides* spp., *Proteus* spp., *Klebsiella* spp., and *Enterobacter* spp.^(4-6)^.

In patients with pylephlebitis, Doppler ultrasound is useful for the characterization of thrombi, portal vein ectasia, collateral venous networks, hepatosplenomegaly, and ascites^(2,4,8-12)^. The diagnostic method of choice is intravenous contrast-enhanced CT, which can reveal gas in the portal venous system (in 18% of cases) and hypodense vascular thrombi. Thrombosis of intrahepatic segments of the portal vein, the superior mesenteric vein, and the splenic mesenteric vein is observed in 39%, 42%, and 12% of cases, respectively, compared with only 2% for the inferior mesenteric vein. Unlike pneumobilia, gas in the portal venous system (hepatic portal venous gas) extends to the hepatic periphery^(2,4,8-12)^.

In cases of pylephlebitis, the most widely used therapy is the combination of anticoagulants and antibiotics. Surgical treatment is reserved for unresponsive cases and for resection of the inflammatory/infectious focus, as well as for drainage of large fluid collections and abscesses^(4-6)^. The reported mortality rates range from 11% to 50%^(2,4-8)^. Complications occur in 20-50% of cases, such complications including hepatic abscesses (in 37%), mesenteric venous infarction, chronic portal vein thrombosis, and portal hypertension^(2,4-8)^.

## References

[1] Fonseca-Neto OCL, Vieira LPF, Miranda AL (2007). Tromboflebite séptica da veia porta secundária
à apendicite. ABCD Arq Bras Cir Dig.

[2] Guimarães RA, Sueth DM, Barros MGCRM (2010). Pileflebite mesentérica secundária à
diverticulite. GED Gastroenterol Endosc Dig.

[3] Gonçalves L, Maio J, Barros MF (2001). Trombose da veia porta. Atitudes. A propósito de caso
clínico. Acta Pediatr Port.

[4] Belhassen-García M, Gomez-Munuera M, Pardo-Lledias J (2014). Pylephlebitis: incidence and prognosis in a tertiary
hospital. Enferm Infecc Microbiol Clin.

[5] Choudhry AJ, Baghdadi YM, Amr MA (2016). Pylephlebitis: a review of 95 cases. J Gastrointest Surg.

[6] Kanellopoulou T, Alexopoulou A, Theodossiades G (2010). Pylephlebitis: an overview of non-cirrhotic cases and factors
related to outcome. Scand J Infect Dis.

[7] Machado MM, Rosa ACF, Mota OM (2006). Ultrasonographic features of portal vein
thrombosis. Radiol Bras.

[8] Balthazar EJ, Gollapudi P (2000). Septic thrombophlebitis of the mesenteric and portal veins: CT
imaging. J Comput Assist Tomogr.

[9] Lee WK, Chang SD, Duddalwar VA (2011). Imaging assessment of congenital and acquired abnormalities of
the portal venous system. Radiographics.

[10] Tandon R, Davidoff A, Worthington MG (2005). Pylephlebitis after CT-guided percutaneous liver
biopsy. AJR Am J Roentgenol.

[11] Muglia VF (2017). Diverticular disease of the colon: evolution of the therapeutic
approach and the role of computed tomography in the evaluation of acute
conditions. Radiol Bras.

[12] Naves AA, D'Ippolito G, Souza LRMF (2017). What radiologists should know about tomographic evaluation of
acute diverticulitis of the colon. Radiol Bras.

